# Lignocellulose degradation in bacteria and fungi: cellulosomes and industrial relevance

**DOI:** 10.3389/fmicb.2025.1583746

**Published:** 2025-04-25

**Authors:** Kuan-Ting Hsin, HueyTyng Lee, Yu-Chun Huang, Guan-Jun Lin, Pei-Yu Lin, Ying-Chung Jimmy Lin, Pao-Yang Chen

**Affiliations:** ^1^Institute of Plant and Microbial Biology, Academia Sinica, Taipei, Taiwan; ^2^Department of Tropical Agriculture and International Cooperation, National Pingtung University of Science and Technology, Pingtung City, Taiwan; ^3^Bioinformatics Program, Taiwan International Graduate Program, National Taiwan University, Taipei, Taiwan; ^4^Bioinformatics Program, Institute of Statistical Science, Taiwan International Graduate Program, Academia Sinica, Taipei, Taiwan; ^5^Genome and Systems Biology Degree Program, National Taiwan University and Academia Sinica, Taipei, Taiwan; ^6^Department of Life Science, National Taiwan University, Taipei, Taiwan; ^7^Institute of Plant Biology, National Taiwan University, Taipei, Taiwan

**Keywords:** cellulosome, lignocellulose, enzymatic degradation, carbohydrate-active enzymes, biomass, biofuel

## Abstract

Lignocellulose biomass is one of the most abundant resources for sustainable biofuels. However, scaling up the biomass-to-biofuels conversion process for widespread usage is still pending. One of the main bottlenecks is the high cost of enzymes used in key process of biomass degradation. Current research efforts are therefore targeted at creative solutions to improve the feasibility of lignocellulosic-degrading enzymes. One way is to engineer multi-enzyme complexes that mimic the bacterial cellulosomal system, known to increase degradation efficiency up to 50-fold when compared to freely-secreted enzymes. However, these designer cellulosomes are instable and less efficient than wild type cellulosomes. In this review, we aim to extensively analyze the current knowledge on the lignocellulosic-degrading enzymes through three aspects. We start by reviewing and comparing sets of enzymes in bacterial and fungal lignocellulose degradation. Next, we focus on the characteristics of cellulosomes in both systems and their feasibility to be engineered. Finally, we highlight three key strategies to enhance enzymatic lignocellulose degradation efficiency: discovering novel lignocellulolytic species and enzymes, bioengineering enzymes for improved thermostability, and structurally optimizing designer cellulosomes. We anticipate these insights to act as resources for the biomass community looking to elevate the usage of lignocellulose as biofuel.

## Graphical abstract

Overview of lignocellulose enzymatic degradation in bacteria and fungi. The left panel shows the degradation enzymes of lignocellulose. Cellulosome is composed of multiple CAZymes and the scaffolding subunit containing scaffoldin, dockerins, cohesins, and carbohydrate-binding module. The middle panel indicates the differences between cellulosomes of bacteria and fungi. The right panel is the potential methods to improve lignocellulose degradation efficiency. DyPs, dye-depolymerizing peroxidases; LMCOs, laccase and laccase-like multi-copper oxidases; LiPs, lignin peroxidases; MnPs, manganese-dependent peroxidases; Lacs, laccases; VPs, versatile peroxidases; CAZymes, carbohydrate-active enzymes; GH, glycoside hydrolase; CE, carbohydrate esterase; AA, auxiliary activity families.

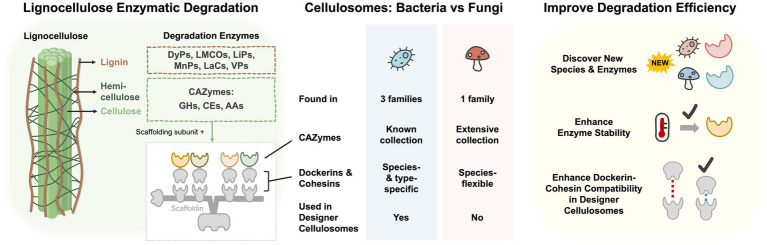

## 1 Introduction

Plant biomass is a resource of renewable energy that is made up of byproducts from agriculture, forestry and the food supply chain. Some examples include wheat straw, sugarcane bagasse, cereal husk and tree bark (Talebnia et al., 2010; Binod et al., 2012; Elmekawy et al., 2013; Jablonsky et al., 2017). With its abundant availability and regenerative nature, plant biomass is a promising candidate to replace fossil fuels as an energy source. Global land biomass was estimated at 1.8 trillion tons in 2018, including 140 billion tons of plant wastes produced annually (Tursi, 2019), altogether enough for world’s annual consumption over 80 times (Anonymous, 2018). However, this resource remains under-exploited. In 2023, biomass energy only made up of about 5% of total energy production globally (Anonymous, 2023).

The main barrier for widespread use of plant biomass energy is the low economic feasibility of upscaling the energy conversion process. Two factors contribute to the cost economics of biomass degradation:

1. The need for pre-treatment, a process to reduce recalcitrance of lignocellulose through thermochemical or physical methods, contributes to about 20% of total cost of production (Yang and Wyman, 2008). Acid hydrolysis and pyrolysis are two common pre-treatments, where concentrated acids and high temperatures ranging from 300 to 800°C are used. Both of these approaches have been proven to be highly efficient, for example, the use of acid hydrolysis pre-treatment prior to enzymatic hydrolysis increases raw poplar wood degradation by 14 times (Antczak et al., 2018). However, these methods impose high costs as they are energy-intensive and may require downstream neutralization of toxic byproducts (Chen et al., 2014).
2. Lignocellulosic-degrading enzymes which perform the key hydrolysis process in breaking down lignocellulose components to yield glucose are highly priced (Singh et al., 2016; Musa and Elnour, 2024). Industrial-grade enzymes have low availability as large amounts of substrates are needed for their production (Bhatia et al., 2021). Although the cost of cellulase enzymes depends on factors like the type of feedstock and enzyme dosage, they can contribute up to 48% of the total cost of producing ethanol from lignocellulose (Liu et al., 2016). In addition, effective biomass degradation requires high concentration of enzymes due to prolonged hydrolysis time (Singhvi and Kim, 2020).

Besides economic feasibility, issues such as substrate specificity of enzymes and the need for optimization of enzymatic cocktails require labor-intensive solutions, further limiting the potential for upscaling biomass usage (Siqueira et al., 2020; Wingren et al., 2003; Hu et al., 2023; Méndez Arias et al., 2016).

### 1.1 The need for cost-effective enzymatic systems

Extensive ongoing research in multiple fields is aimed at designing a cost-effective enzymatic system which requires minimum pre-treatment and lowering the costs of degrading enzymes. For example, developing new pre-treatment methods for recalcitrant components (Wang et al., 2013), simplifying enzyme purification methods and logistics (Takimura et al., 2013; Nhim et al., 2024), and modification of enzyme properties such as activity and thermostability through protein engineering methods (Bloom et al., 2005).

The graphical abstract depicts three aspects of lignocellulosic-degrading enzymes that we are focusing on in this review. First, we characterized the sets of enzymes naturally involved in bacterial and fungal lignocellulose degradation as shown in the left panel of the graphical abstract. In the middle panel, we explore the characteristics of a highly-efficient multi-enzyme complex termed cellulosome in bacterial and fungal systems, and their feasibility to be engineered. Thirdly, as shown in the right panel, we describe three main directions in enhancing enzymatic degradation efficiency in biomass usage.

## 2 Enzymatic degradation of lignocellulose

Lignocellulosic biomass consists of three main components, cellulose (60%), hemicellulose (17–32%) and lignin (10–25%) (Payne et al., 2015; Sankaran et al., 2021; Akinosho et al., 2014). Cellulose is composed of a linear polymer chain of 7,000–15,000 glucoses connected by β-1-4-glycosidic bonds in a cross-linked structure, which prevents enzyme access (Rosenau et al., 2018), forming one of the most stable polymers on earth. Hemicellulose is a branched heteropolysaccharide composed of various sugar monomers, such as xylose, arabinose, mannose, and glucose, and its structure varies across plant species (Gao et al., 2023; Paz-Cedeno et al., 2022). It exhibits a lower degree of polymerization and reduced molecular weight in comparison to cellulose. Hemicellulose functions as a linking agent between lignin and cellulose fibers, as well as a physical barrier that obstructs access to cellulose, contributing to increased rigidity within the composite structure (Syguła et al., 2024). Lignin, the compound that contributes to biomass recalcitrance, i.e., degree of resistance against degradation, consists of cross-linked phenyl propane polymers (Gibson, 2012). It contributes to cell wall integrity and forms an outer barrier for degrading enzymes to access cellulose and hemicellulose (Boerjan et al., 2003).

The enzymatic degradation of lignocellulose can be achieved by diverse bacterial and fungal species, each exhibiting differing efficiencies and enzyme sets. Table 1 summarizes the functions and characteristics of cellulose, hemicellulose and lignin, as well as whether bacterial and fungal enzymatic degradation is possible. Enzymes that are involved in breaking down complex carbohydrates and polysaccharides into smaller products are known as carbohydrate-active enzymes (CAZymes), which include Glycoside Hydrolase (GH), Carbohydrate Esterase (CE) and Auxiliary Activity (AA) families (Kameshwar et al., 2019). These enzymes act in tandem to target different components of lignocellulose. For example, the degradation of hemicellulose requires backbone-cleaving GHs and ancillary enzymes that attack the functional groups.

**Table 1:** Features of the three main components in lignocellulose.

Features	Cellulose	Hemicellulose	Lignin
Content in lignocellulose	60%	17–32%	10–25%
Function	Predominant component in cell wall	Physical barrier to accessibility of enzymes	Confers hydrophobicity and structural rigidity
Characteristics	Linear polymer of glucoses linked by β-1-4 glycosidic bonds	Branched polymer of various monosaccharides	Network of heterogeneous, alkyl-aromatic polymer
Recalcitrance	High	Low	High
Bacterial degradation	Yes	Yes	Limited
Fungal degradation	Yes	Yes	Yes

## 3 Cellulose and hemicellulose degradation

Cellulose and hemicellulose degrading bacteria can be found in the environment as well as in the gastrointestinal system of ruminant animals (Weimer, 2022; Datta, 2024). Both aerobic and anaerobic bacteria are capable of degrading cellulose and hemicellulose. For fungi, they are mainly identified within Ascomycetes and Basidiomycetes (Li et al., 2022). These fungi are divided into brown-rot, white-rot, and soft-rot fungi based on their ability to decompose each type of lignocellulose component (Fukasawa, 2021). Here we discuss and compare the degradation mechanisms of cellulose and hemicellulose in bacteria and fungi.

### 3.1 Bacteria and fungi share similar degradation enzymes

The degradation of cellulose is achieved by GHs, which function to break the glycosidic linkage between two carbohydrates. There are three types of GH families characterized by their modes of activity and location of cleavage (Akinosho et al., 2014; Datta, 2024; Bhardwaj et al., 2019):

1. Endoglucanases cleave β-1,4-glycosidic bonds from the internal structure and are more active on the soluble region of the substrate. GH5 to GH12, GH26, GH44, GH45, GH48, GH51, GH74, and GH124 are regarded as endoglucanases (Alnoch et al., 2023).
2. Exoglucanases cleave bonds from the terminals of substrates and release cellobiose molecules. GH3, GH43 and few members from other GH families belong to this category (Hirano et al., 2016).
3. β-glucosidases are responsible for degrading the cellobiose released from endoglucanases and exoglucanases. β-glucosidases remove the non-reducing end glucosyl residues from saccharides and glycosides to produce glucose (Ketudat Cairns and Esen, 2010; Puchart et al., 2023). GH1-GH3, GH5, GH30, GH39 and GH116 members are key β-glucosidases.

Due to the diverse nature of hemicellulose composition, multiple sets of enzymes are involved in its degradation. To degrade xylans, the xylanase endoenzymes, such as those from GH10, function by hydrolyzing the β-D-xylopyranosyl bonds to release xylobiose, xylooligosaccharides, and xylose (Puchart et al., 2021). As for mannans, the β-mannanase, β-mannosidase, acetyl mannan esterase, β-glucosidase and α-galactosidase belonging to GH1, GH2, GH3, GH5, GH26, GH27, and GH113 (Dawood and Ma, 2020) are involved. The GH74, GH29 and GH95 members exhibit the xyloglucan-specific endo-β-1,4-glucanase activity to degrade the backbone chain of xyloglucan (Zerillo et al., 2013).

CEs and AAs are accessory enzymes that facilitate the actions of GHs in degrading cellulose and hemicellulose. CEs target pectin oligosaccharides and arabinoxylans to cleave ester linkages and release acyl or alkyl, while AA9 to AA17 families consist of lytic polysaccharide monooxygenases (LPMOs) that cleave the crystalline polysaccharide chain of cellulose and other carbohydrates via direct oxidative reactions (Ward et al., 2020).

In general, bacteria and fungi share similar sets of enzymes listed above to degrade cellulose and hemicellulose (Haitjema et al., 2017; Floudas et al., 2012). However, certain groups of fungi possess greater variety of enzymes than others, therefore contributing to different degree of degradation ability. For example, a genome-wide search revealed that white-rot fungi generally possess more GHs compared to brown-rot fungi, suggesting a divergence in the GH family between these two types of fungi (Riley et al., 2014). White-rot fungi encode GH6 and GH7 enzymes that can degrade crystalline cellulose, which are missing in brown-rot fungi likely due to gene loss events (Floudas et al., 2012).

In addition, endoglucanases of certain fungal species have been shown to be more effective in targeting amorphous domains, most likely because fungi species have more “retaining” type endoglucanases such as GH7, GH5 and GH12 (Bharadwaj et al., 2020) than the bacterial species. “Retaining” type endoglucanases are known to target cellulose domains that are disordered and therefore have lowered recalcitrance (Ioelovich, 2021; Wang et al., 2016).

## 4 Lignin degradation

Lignin degradation involves the breaking down of alkyl-aromatic polymers into simpler compounds. It occurs in two steps, depolymerization and aromatic ring cleavage. Depolymerization occurs via the oxidative degradation mechanism, while the aromatic ring cleavage step involves long-range electron transfer process and phenolic moieties oxidation (Higuchi, 2004). In both bacteria and fungi, the cleavage of the aromatic ring is catalyzed by non-heme iron-containing intradiol and extradiol dioxygenases (Harayama and Rekik, 1989; Semana and Powlowski, 2019). Intradiol dioxygenases cleave catecholic substrates between the two hydroxyl groups using a non-heme Fe (III) center, while extradiol dioxygenases cleave catecholic substrates adjacent to the hydroxyl groups and typically contain a non-heme Fe (II) center.

Both bacterial and fungal species exhibit lignin degradation ability, for example the fungus *Aspergillus flavus* has a lignin degradation rate of 44.6% within 3 days under optimized conditions (Li et al., 2020) while the bacterium *Bacillus cereus* shows a lignin degradation rate of 89% (Kumar et al., 2022). The enzymes involved in lignin degradation of fungi and bacteria are presented in the following sections.

### 4.1 Fungi possess a diverse set of lignin degradation enzymes

The white-rot fungi are well-known to degrade the lignin. A diverse set of lignin degradation enzymes is found in white-rot fungi, including dye-depolymerizing peroxidases (DyPs), laccase and laccase-like multi-copper oxidases (LMCOs), lignin peroxidases (LiP), manganese-dependent peroxidases (MnP), laccases (LaC), versatile peroxidases (VP) and unspecific peroxygenases (UPO) (Yadav et al., 2022). DyPs belong to a superfamily of heme peroxidases (Sugano, 2009), whereas LMCOs belong to the multicopper oxidase superfamily (Leonowicz et al., 2001). Both DyPs and LCMOs form two major groups of lignin-degrading enzymes in fungi.

DyPs exhibit broad substrate specificity, oxidizing typical peroxidase substrates such as 2,2′-Azino-bis(3-ethylbenzothiazoline-6-sulfonic acid) (ABTS), 2,6-dimethoxyphenol (DMP) and various dyes, and potentially demonstrate additional hydrolase or oxygenase activities (Sugano, 2009). The LMCOs demonstrate oxidase activity, facilitating the oxidation of diverse phenolic compounds as well as certain non-phenolic substrates, while simultaneously reducing molecular oxygen to water (Ausec et al., 2011). The LiPs degrade the aromatic compounds and phenolic compounds in lignin with the presence of H_2_O_2_ via oxidation processes (Kumar and Chandra, 2020). MnP facilitates the oxidation of Mn^2+^ to Mn^3+^ using hydrogen peroxide, forming a Mn^2+^/Mn^3+^ redox pair. In the presence of a chelating agent, such as oxalate or malate, the Mn^3+^ ion is released from the enzyme’s active site (Kumar and Chandra, 2020; Janusz et al., 2017). These chelators stabilize Mn^3+^, preventing its disproportionation into Mn^2+^ and insoluble Mn^4+^. The stabilized Mn^3+^ complex can then diffuse into the lignified cell wall, where it oxidizes phenolic and nonphenolic lignin components. Versatile peroxidases possess a unique substrate specificity that combines the characteristics of LiP and MnP (Yadav et al., 2022). Unlike LiPs and MnPs, VPs can oxidize a broader range of phenolic and nonphenolic substrates, including veratryl alcohol, methoxybenzenes, and various lignin model compounds. UPOs are multifunctional oxidoreductases with wide substrate versatility as they are able to act both as oxygen donors and reductants (Ullrich et al., 2004; Pandya et al., 2014). This dual-functionality enables UPOs to emerge as research targets for process development (Tieves et al., 2019).

Some brown-rot fungi, like *Gloeophyllum trabeum* also exhibit lignin degradation ability (Arimoto et al., 2015). Though both brown-rot and white-rot fungi break down lignin by releasing acyl or alkyl groups (Wilhelm et al., 2019; de Gonzalo et al., 2016; Grgas et al., 2023; Christopher et al., 2014), their mechanisms differ significantly (Janusz et al., 2017; Cragg et al., 2015; Embacher et al., 2023). Brown-rot fungi use a non-enzymatic mechanism known as the Fenton reaction, an energy-saving process and relies on Fe (II) ions and hydrogen peroxide (Yadav et al., 2022; Embacher et al., 2023). This reaction reduces Fe (III) to Fe (II) in plant cells, producing-OH radicals that depolymerize lignin polymers (Yadav et al., 2022; Embacher et al., 2023) and break down cellulose and hemicellulose, making them more accessible to other lignocellulose degradation enzymes. Some Ascomycetes, including soft-rot fungi, secrete oxidative enzymes such as laccases and peroxidases, which help degrade lignin by oxidizing its phenolic units and breaking down its structure using hydrogen peroxide, respectively. For example, *Podospora anserina* demonstrates its ability to break down lignin through enhanced synthesis of laccases (Ferrari et al., 2021; van Erven et al., 2020).

### 4.2 Bacterial lignin-degrading enzymes: low efficiency, high adaptability

Lignin-degrading enzymes have been identified genetically (Janusz et al., 2017; Bugg et al., 2011) and isolated from many bacteria species. However, the degradation strategies in bacteria are less understood, and are believed to be generally less efficient compared to fungi (He et al., 2017). Bacteria capable of lignin modification and degradation belong to class Actinomycetes which mainly live in the soil (Bugg et al., 2011), as well as Gammaproteobacteria and Alphaproteobacteria (Xu et al., 2019). Only two classes of lignin-degrading enzymes are identified in bacteria, DyPs and LMCOs (Kamimura et al., 2017; Levy-Booth et al., 2022; Wilhelm et al., 2019). Bacterial laccases and DyPs have relatively lower oxidizing power than fungal enzymes, and therefore target lignin polymers of lower molecular weight (de Gonzalo et al., 2016).

Despite their low efficiency, bacterial lignin-degrading enzymes can function in a broader range of environmental conditions (Grgas et al., 2023). With better thermal stability and higher optimal pH (Levy-Booth et al., 2022; Christopher et al., 2014), bacterial laccases are attractive candidates for industrial usage. Another advantage unique to bacterial lignin-degradation is their capability of modifying or converting lignin into valuable bioproducts. Instead of rapidly degrading it in order to access cellulose and hemicellulose, bacteria utilize lignin as a substrate. After lignin monomers are converted into phenolic compounds, aromatic ring cleavage will occur for cellular uptake while producing bioproducts that are of industrial interest such as muconic acid and polyhydroxyalkanoates (Li and Zheng, 2020).

As examples, Table 2 details the extensive sets of carbohydrate-active enzyme families identified in four bacteria and seven representative fungal species.

**Table 2:** Carbohydrate-active enzymes identified from microorganism representatives.

Species	**Enzyme types**											
	Glycoside hydrolases (GH)								Auxiliary activity	Carbohydrate esterase (CE)	Polysaccharide lyase (PL)	Others
	Refs	β	x, β	n, x	n, x, β	n	x	Uncategorised				
*A. thermophyllum* (CB)	Wei et al. (2014)	2, 30, 81		9, 48	5	8, 10, 11, 26, 74, 124, 16, 53	27, 43			C3, C12	PL1, PL11	C9, 11A, 12A, 18A
*T. stercorarium* (CB)	Broeker et al. (2018)	2, 39	3	9		10, 11, 26, 51, 53	28, 31, 43	29, 38, 67, 78, 88, 95, 115, 127				
*C. saccharoperbutylacetonicum* (CB)	Huckfeldt and Schmidt (2015)	30		9, 48	5	44, 8, 10, 11, 26, 74, 98	43			C12	PL1, PL11	Lip, Pep
*R. champanellensis* (CB)	Tanaka et al. (1988)	30		9, 48	5	44, 8, 10, 11, 26, 74, 98	43			C12	PL1, PL11	Lip, Pep
*A. robustus* (CF)	Brown et al. (2023)	1, 2, 30, 39	3	9, 48	5	45, 6, 8, 10, 11, 26, 13, 16, 114	18, 31, 43	95, 115				
*P. finnis* (CF)	Haitjema et al. (2014)	1, 2, 30, 39	3	9, 48	5	45, 6, 8, 10, 11, 26, 13, 16, 53, 114	18, 31, 43	38, 95, 115		C1, C4, C8, C12, C15	PL1	
*C. subvermispora* (FF)	Fernandez-Fueyo et al. (2012)				5	6, 7, 12				C1, C8, C15, C16		
*P. chrysosporium* (FF)	Fernandez-Fueyo et al. (2012)		3		5	6, 7, 10, 11, 12, 74	28, 43		AA9	C1, C8, C15, C16		
*T. versicolor* (FF)	Floudas et al. (2012)		3		5	6, 7, 10, 12, 74	28, 43	115, 125	AA9	C4, C8, C15, C16		
*W. cocos* (FF)	Floudas et al. (2012) and Gaskell et al. (2016)	1, 2, 79	3		5	10, 12, 16	18, 28, 43, 131	78	AA9	C1, C8		
*G. trabeum* (FF)	Floudas et al. (2012) and Umezawa et al. (2020)	1, 81	3		5	10, 12	28			C3, C12	PL1, PL11	11A, 12A, 18A

CB, Cellulosome-producing bacteria; CF, Cellulosome-producing fungi; FF, Free enzyme-producing fungi; Numbers represent GH families. n, endoglucanase; x, exoglucanase; β, β-glucosidases; C9, cellobiohydrolase 9; Lip, lipase; Pep, peptidase; 18A, chitinolytic enzyme 18A; 12A, rhamnogalacturonan acetyl esterase 12A; 11A, rhamnogalacturonan lyase 11A; Refs, references.

## 5 The assembly of cellulosome enhances degradation efficiency

**Figure 1:**
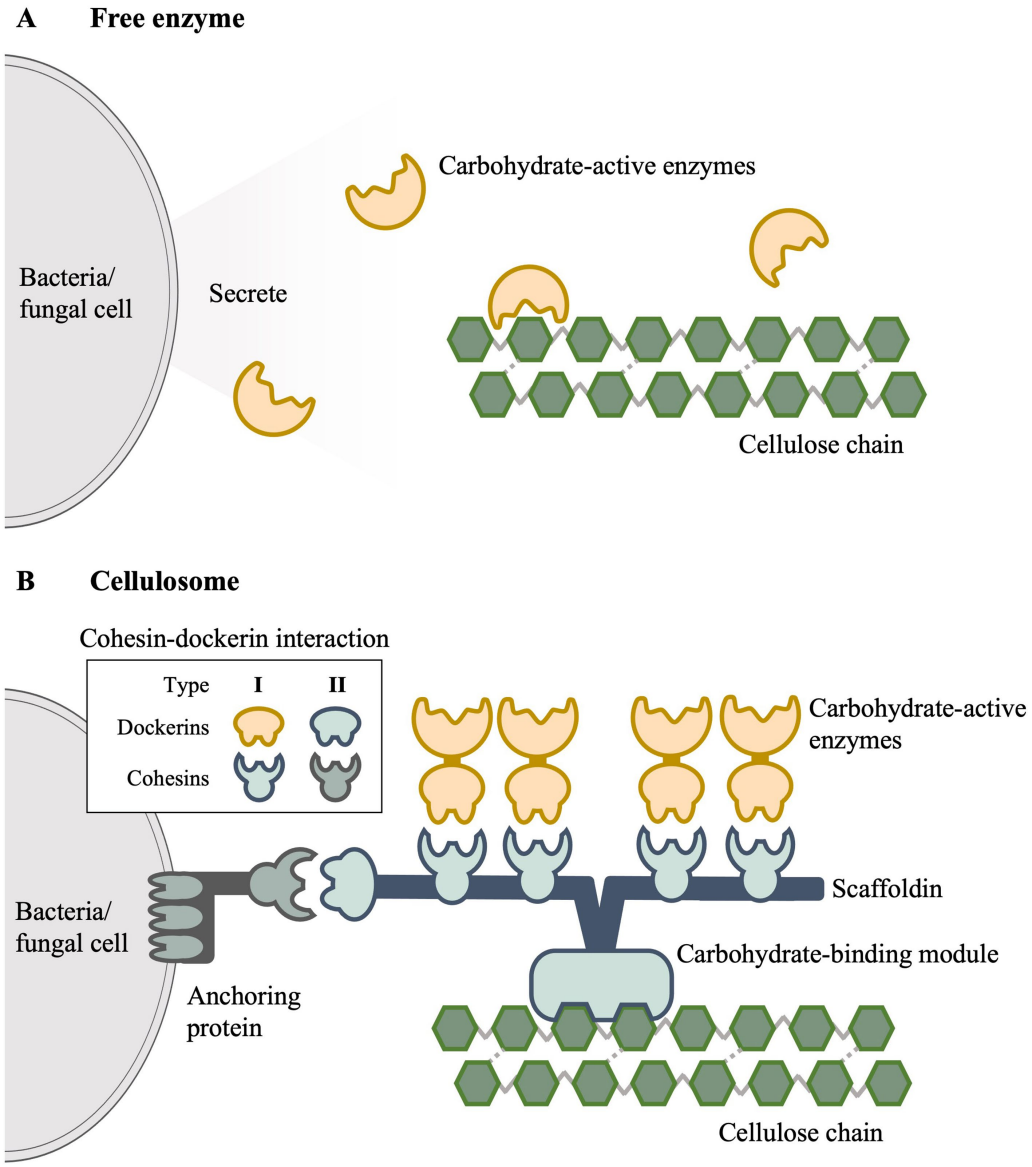
Overview of enzymatic mechanisms of lignocellulose degradation. **(A)** Free enzyme mechanism. **(B)** Cellulosome.

The enzymes described above can either be freely-secreted or assembled into a multi-enzyme complex known as cellulosome. Majority of the lignocellulolytic bacteria and fungi that are aerobic belong to the freely-secreting category. As shown in Figure 1A, secreted enzymes diffuse as individual catalytic units and function independently to degrade lignocellulose (Cragg et al., 2015). *Trichoderma reesei* and *Aspergillus niger* are the two main fungi species that are being extensively exploited for the production of cellulolytic cocktails (Xu et al., 2009). Bacterial strains are generally less favored in industrial settings due to their lower secretion capacity although species such as *Streptomyces* have been identified as versatile candidates (Kashiwagi et al., 2017).

On the other hand, the cellulosome, first discovered in *Acetivibrio thermocellum* (Bayer et al., 2004; Lamed and Bayer, 1988), is a multiprotein complex in which lignocellulolytic enzymes are tethered together for enhanced efficient degradation and systematic hydrolytic activity (Artzi et al., 2017). A simplified cellulosome is depicted in Figure 1B, where it is predominantly situated on the cell wall surface of microorganisms with enzymes attached to a large non-catalytic subunit known as scaffoldin. This building-block-like structure is assembled through a flexible mechanism involving specialized domains called cohesins and dockerins. The type I cohesin-dockerin interaction allows binding of carbohydrate-active enzymes to scaffoldin (Fan et al., 2012), while type II interaction allows the scaffoldin to anchor to the cell surface (Doi et al., 2003). The carbohydrate-binding module (CBM) serves to attach the cellulosome structure to the cellulose substrate (Akinosho et al., 2014; Doi et al., 2003), while the connection of cellulosome to the cell surface is facilitated by surface (S)-layer proteins (Bayer et al., 2008; Fontes and Gilbert, 2010).

The efficiency of bacterial cellulosome has been demonstrated through comparisons of cellulose hydrolysis rate, with *A. thermocellum* achieving a 50-fold increase over free enzymes in *T. ressei* (Demain et al., 2005) and a 15-fold decrease when *A. thermocellum*’s cellulosome formation is impaired (Zverlov Vladimir et al., 2008). It has been proposed that the spatial enzyme proximity offered by cellulosome structure enhances synergistic interactions between catalytic units (Bayer et al., 2004), while anchoring to the cellulosome increases enzyme stability (Fontes and Gilbert, 2010). Cellulosomes can be found in anaerobic bacteria of the Clostridia class and Neocallimastigomycetes fungi (Artzi et al., 2017; Doi et al., 2003; Xu et al., 2003; Xu et al., 2004; Sabathé et al., 2002; Zhivin et al., 2017; Morrison and Miron, 2000; Moraïs et al., 2016; Ding et al., 2001; Cai et al., 2010; Levi Hevroni et al., 2020; Ben David et al., 2015). While none of the cellulosomal-producing species are currently used in industrial applications, they are key research targets in optimizing the biofuel production process (Vodovnik and Lindic, 2025).

### 5.1 The building blocks of cellulosomes: cohesins and dockerins

Components of cellulosomes are held together by high-affinity interactions (Pagès et al., 1997; Fierobe et al., 2002) between cohesins and dockerins. Three types of cohesin-dockerin interactions were identified so far based on sequence similarity: type I which is responsible for attaching enzymes to scaffoldin, type II which attaches scaffoldin to the cell, and type III which consists of those that are sequentially divergent from the other types (Karpol et al., 2013). Note that this classification is based on bacterial cellulosomes, and the more recently discovered fungal cohesins and dockerins are currently unclassified (Alves et al., 2021).

Cohesin-dockerin has type-and species-specificity, where cohesins of Type I do not interact with dockerins of Type II (Fontes and Gilbert, 2010; Miras et al., 2002; Schaeffer et al., 2002), and cohesins of one species do not interact with dockerins of another species (Fendri et al., 2009). Analysis of binding sites showed that low sequence similarities and differences in structural site topology explain these specificities in cohesins and dockerins (Carvalho et al., 2005). The combination of strict species-and type-specificity with module-promiscuity enables accurate assembly of cellulosome with enough heterogeneity of enzyme content, a factor that has been identified to increase degradation efficiency (Fendri et al., 2009).

As cellulosome is an inherently dynamic structure (Barth et al., 2018), the precise arrangement of the components, including cohesins and dockerins, remains poorly understood in most species. Firstly, the amino acid residues that are responsible for binding specificity and promiscuity as described above is not well understood, as mutational studies only attained conclusive results in limited species (Slutzki et al., 2015). Secondly, cohesins and dockerins have been found to play a role in maintaining the plasticity and stability of the whole cellulosome, but the mechanism remains unclear. Alternative binding modes have been found in certain types of cohesin (Alves et al., 2021; Carvalho et al., 2007) which may enable flexibility in the spatial organization of the scaffoldin subunits (Lamote et al., 2023). These binding modes had been hypothesized to be enzymatically regulated by an intramolecular structure located on dockerins. Also, the precise spatial arrangement of enzymatic subunits is found to rely on cohesin-cohesin interactions (Barth et al., 2018). These findings suggest that cohesins and dockerins are the basis of the regulation of cellulosome stability and plasticity, but quantitative evidence to these hypotheses is still lacking (Lamote et al., 2023).

Thorough understanding of the regulation and organization of cohesin-dockerin interactions may be able to improve the process of lignocellulose degradation, such as development of methods to manipulate the specificity of cohesin-dockerin complexes, as well as to enhance stability and flexibility of artificial designer cellulosomes.

### 5.2 Characterization of cellulosomes based on bacterial systems

The architecture of cellulosomes can vary from simple to complex, depending on the amount of interacting scaffoldins and the intricacy of the complex (Bayer et al., 2004). A simple cellulosome is composed of a single scaffoldin that contains up to nine enzymatic subunits, according to the number of cohesin modules on the scaffoldin, like in *Clostridium josui* (Ichikawa et al., 2014). Highly structured cellulosomes contain several scaffoldins and many enzymes. For example, eight scaffoldins and up to 63 enzymes are identified from *A. thermocellum* (Fontes and Gilbert, 2010).

Cellulosomes display selective enzyme synthesis based on the substrate. For instance, when grown on differently pretreated yellow poplar and pine-made pulp, *A. thermocellum*’s cellulosomes show varied enzyme compositions (Wei et al., 2014; Leis et al., 2018). Pine pulp, with its higher lignin content, leads to the production of enzymes like xylanase and mannanase for lignin breakdown, while pretreated yellow poplar prompts the synthesis of endoglucanases, exoglucanases, and β-glucosidases (Leis et al., 2018). Similarly, *Clostridium cellulovorans* produces different sets of enzymes, when grown on cellulose, xylan, galactomannan and pectin (Aburaya et al., 2019).

Current understandings of cellulosomes are mainly derived from bacterial species, as only limited fungal cellulosomes are discovered to date. Table 3 lists 22 bacteria and 4 fungi species that were commonly reported in the literature to produce cellulosomes. Cellulosome is presently exclusive to anaerobic bacteria and fungi of Clostridia and Neocallimastigomycetes members (Artzi et al., 2017; Doi et al., 2003; Xu et al., 2003; Xu et al., 2004; Sabathé et al., 2002; Zhivin et al., 2017; Morrison and Miron, 2000; Moraïs et al., 2016; Ding et al., 2001; Cai et al., 2010; Levi Hevroni et al., 2020; Ben David et al., 2015). Class Clostridia consists of anaerobic bacteria that mainly colonize the human gastrointestinal system and soil, whereas Neocallimastigomycetes is a phylum of anaerobic fungi that colonizes the herbivore gastrointestinal system. The strict anaerobic environment and limited energy sources of anaerobic bacteria are two factors that are hypothesized to impose selective pressures for the evolution of cellulosomes. This adaptation allows for efficient lignocellulosic degradation to obtain cellular energy (Fontes and Gilbert, 2010).

**Table 3:** Cellulosome-producing bacteria and fungi.

Cellulosome-producing bacteria			
Highly structured cellulosome			
Class: Clostridia	Genus: *Acetivibrio*	*Acetivibrio cellulolyticus*	Zhivin et al. (2017)
		*Acetivibrio alkalicellulosi*	Zhivin et al. (2017) and Karpol et al. (2013)
		*Acetivibrio straminisolvens*	Zhivin et al. (2017) Alves et al. (2021)
		*Acetivibrio clariflavum*	Zhivin et al. (2017) and Fendri et al. (2009)
		*Acetivibrio saccinicola*	Miras et al. (2002)
		*Acetivibrio thermocellum*	Zhivin et al. (2017)
	Genus: *Pseudobacteroides*	*Pseudobacteroides cellulosolvens*	Schaeffer et al. (2002) and Pages et al. (1999)
	Genus: *Ruminococcus*	*Ruminococcus albus*	Ding et al. (2001)
		*Ruminococcus champanellensis*	Zhivin et al. (2017)
		*Ruminococcus flavefaciens*	Israeli-Ruimy et al. (2017)
	Genus: *Ruminiclostridium*	*Ruminiclostridium hermifermentans*	Rettenmaier et al. (2019)
		*Ruminiclostridium sufflavum*	Rettenmaier et al. (2019)
Simple cellulosome			
Class: Clostridia	Genus: *Clostridium*	*Clostridium acetobutylicum*	Artzi et al. (2017)
		*Clostridium cellobioparum*	Artzi et al. (2017)
		*Clostridium cellulovorans*	Artzi et al. (2017)
		*Clostridium saccharoperbutylacetonicum*	Artzi et al. (2017)
	Genus: *Ruminiclostridium*	*Ruminiclostridium cellobioparum*	Artzi et al. (2017)
		*Ruminiclostridium cellulolyticum*	Artzi et al. (2017)
		*Ruminiclostridium papyrosolvens*	Artzi et al. (2017)
		*Ruminiclostridium cellobioparum sub*sp. *termitidis*	Artzi et al. (2017)
		*Ruminiclostridium josui*	Artzi et al. (2017)
	Genus *Herbinix*	*Herbinix luporum*	Koeck et al. (2015)
Cellulosome-producing fungi			
Highly structured cellulosome			
Class: Neocallimastigomycetes	Genus: *Neocallimastix*	*Neocallimastix californiae*	Haitjema et al. (2017)
	Genus: *Orpinomyces*	*Orpinomyces* sp. C1A	Haitjema et al. (2017)
	Genus: *Piromyces*	*Pyromyces sp.* E2	Haitjema et al. (2017)
		*Pyromyces finnis*	Haitjema et al. (2017)

### 5.3 Fungal cellulosomes are poorly characterized due to genetic and isolation challenges

Fungal cellulosomes remain poorly understood due to the limited number of identified cellulosome-producing fungi and the low genetic similarity of their cellulosomal genes to bacterial counterparts (Youssef Noha et al., 2013). This means that the knowledge from bacterial cellulosomes might not be directly transferable onto fungal cellulosomes. To date, fungal cellulosomes have been isolated from anaerobic fungi residing in gastrointestinal systems of herbivores (Haitjema et al., 2017; Lillington et al., 2021; Brown et al., 2023; Gomez-Flores et al., 2017). Despite the early 20th-century discovery of fungal cellulosomes in the anaerobic rumen fungus *Neocallimastix frontalis* (Wilson and Wood, 1992), it was not until recently that their biological characteristics and gene-level components were unveiled (Haitjema et al., 2017; Lillington et al., 2021; Brown et al., 2023). This delay was due to the challenges in isolating these organisms, attributed to their anaerobic nature and complex nutritional needs (Haitjema et al., 2014). The biological features of the fungal cellulosome were initially explored through the herbivore gut-isolated *Piromyces finnis* (Lillington et al., 2021) and the presence of dockerin domains, partial ScaA scaffolding, and GH48 indicate that fungal cellulosomes consist of similar compounds as bacterial cellulosomes.

### 5.4 Key differences between bacteria and fungi cellulosomes

There are two key differences in cellulosomes between bacteria and fungi:

1. The genetic sequences of fungal dockerin and cohesin domains have no similarity to bacterial counterparts (Haitjema et al., 2017). The fungal dockerins form tandem repeats, which are believed to enhance cellulosome binding more than singular, non-repeated domains (Raghothama et al., 2001). Contrary to the precise dockerin-cohesin interaction observed in bacteria (Bayer et al., 2004), fungal versions have low species-specificity. Dockerins from one fungal species can attach to cellulosome components of another (Fanutti et al., 1995), suggesting the potential for various fungal species within the gut to aid in cellulosome structure formation, thereby boosting its efficiency (Haitjema et al., 2017). This cross-binding capability, so far confirmed only in species from the early-diverging fungal phylum Neocallimastigomycota, suggests a possibly widespread conservation of the scaffoldin and dockerin/cohesin system.
2. The second key difference is that fungi with cellulosomes possess the most extensive collection of carbohydrate-active enzymes discovered to date (Solomon et al., 2016). Of all the dockerin-domain-containing proteins in five strains of anaerobic fungi, 13% are GH enzymes that are absent in bacterial cellulosomes (Haitjema et al., 2017), therefore contributing enzymatic functions that are unique to fungi. For example, using β-glucosidases GH3, fungal cellulosome is able to convert cellulose into monosaccharides, instead of the more complex oligosaccharides produced by bacterial cellulosome (Steenbakkers et al., 2003). Fungal cellulosomes also prefer to degrade more recalcitrant lignocellulose components compared to bacterial cellulosomes (Hagen et al., 2021).

Even though the understanding for fungal cellulosome is still based on pieces of evidence, unique features that stood out from bacterial cellulosome may suggest fungal cellulosome to be a potential candidate for industrial applications.

## 6 Ways to improve lignocellulose degradation for industrial applications

Currently, most industrial applications depend on enzymatic cocktails derived from aerobic fungi such as *T. reesei* and *Aspergillus* sp. (Seppälä et al., 2017). The significant expense of these mixtures is a major barrier to making biofuels economically viable (Klein-Marcuschamer et al., 2012). Moreover, optimizing these mixtures based on the substrate is necessary, as the generic “one-size-fits-all” approach has been proven ineffective (Chylenski et al., 2017).

Bioengineering and genomic advancement can help to improve the process by shortening the reaction time of enzymes, reducing the enzyme concentration needed, and enhancing enzyme stability in extreme heat or pH conditions. Here we discuss three main focuses in optimizing lignocellulose degradation for industrial applications.

### 6.1 Discovery of novel lignocellulolytic species and enzymes

With more and more species being isolated and sequenced, genomic (such as NCBI) and proteomic [such as CAZy (Drula et al., 2022)] databases are exponentially increasing in size. Discovering and adding novel lignocellulose-degrading species and enzymes in various ecosystems to the existing repertoire is a highly valuable effort to improve lignocellulose degradation for industrial use.

One example is the aforementioned gut anaerobic fungi. While genomic studies of these gut fungi species showed that their enzyme repertoires possess four times more carbohydrate-active enzymes than fungi that utilize free enzyme mechanisms, a large portion of open reading frames in their genomes remain unannotated (Seppälä et al., 2017), therefore opening the possibility of discovering novel, and efficient enzymes. Similar findings have been obtained through metagenomic analysis. For example, highly complexed microbial communities with a diversified set of cellulases and accessory enzymes were found in three naturally-degraded energy crops (Montella et al., 2017). A recent genomic scan of bacterial genomes for scaffoldins, cohesins, dockerins and enzymes revealed 9 new bacterial species that are likely to produce simple cellulosomes (Minor et al., 2024).

### 6.2 Engineering lignocellulolytic enzymes for thermostability

Enzyme engineering is a common practice to modify enzymatic properties through altering the amino acid sequences (Bloom et al., 2005). One of the more popular procedures is through random mutagenesis directed evolution, where a target gene in a microorganism is randomly mutated followed by high-throughput screening approaches (Liu et al., 2009). For example, thermostability is one of the highly desired traits, as besides maintaining reactivity in high temperature, thermostable enzymes can be recycled more efficiently (Skovgaard and Jorgensen, 2013). A quadruple mutant of *A. thermocellum* endoglucanase Cel8A was produced through consensus-guided mutagenesis and molecular dynamics simulation, where its hydrolysis activity was increased by 14-fold at 85°C compared to wild type enzyme (Anbar et al., 2012). Similarly, thermostable exoglucanase (Smith et al., 2012) and β-glucosidase (Yoav et al., 2019) were also successfully generated in the recent years. These results demonstrated bioengineering as a reliable tool in producing enzymes of desired qualities for industrial use.

### 6.3 Improving structural stability of designer cellulosomes

Designer cellulosomes constructed with bacteria *A. thermocellum* and *A. cellulolyticus* have shown to be two to three times more effective at breaking down crystalline lignocellulose than free enzymes (Fierobe et al., 2002; Morais et al., 2011; Tsai et al., 2013; Gefen et al., 2012). Designer cellulosomes offer a key advantage by enabling the integration of diverse enzymes from multiple species, including both cellulosomal and non-cellulosomal enzymes, which has been shown to increase degradation efficiencies. Reconstituted *A. thermocellum* cellulosome with “external” enzymes that is from other species are more effective in degrading crystalline cellulosome (Hirano et al., 2016). Incorporation of non-cellulosomal enzymes such as LPMOs and expansins also showed similar effects (Chen et al., 2016; Arfi et al., 2014). Cross-species combination of cellulosomal enzymes with dockerin domains has also been shown to have a positive effect on degradation efficiency as glucose production is enhanced when β-glucosidases from *A. thermocellum* was tagged with the dockerin from *Ruminococcus flavefaciens* (Tsai et al., 2009). The benefit of cross-species combination of enzymes is also proven in fungi, as different types of fungi follow a succession in natural wood decay. The process begins with early colonizers consuming residual sap carbohydrates, followed by intermediate decay by brown-rot and white-rot fungi, and ends with soft-rot fungi thriving in nutrient-rich conditions, ultimately leading to lignin-degraded wood dominated by soft-rot fungi (Fukasawa, 2021; Fukasawa et al., 2009). This shows that in order to effectively degrade the lignocellulose, a combination of species is required. Structural stability of designer cellulosomes can be enhanced via cohesins-dockerins affinity, and improving the thermostability of other components.

#### 6.3.1 Improving the compatibility between cohesins and dockerins

Designer cellulosomes have issues of low mechanical stability, which is mainly caused by the compatibility of selected cohesins and dockerins (Levasseur et al., 2004). It is necessary to ensure that the selected cohesin-dockerin pair is only able to interact with each other and not with any other modules that are incorporated in the design (Lamote et al., 2023). However, often times the molecular characterization of cohesins and dockerins available are not thorough enough to verify their specificities. For example, differing concentration and pH have been shown to affect binding specificities. The species-specific interaction between cohesins and dockerins typically occurs at low concentrations; however, at higher concentrations of dockerin-containing enzymes, dockerins can also interact with non-cognate but closely related cohesin domains (Barak et al., 2005). In addition, pH value is identified as a factor affecting cohesins-dockerins affinity, which regulate the amounts of protein complex and the structural stability of designer cellulosome (Barbosa et al., 2010; Duarte et al., 2023; Yao et al., 2020).

To optimize the selection of cohesin-dockerin pairs, deciphering the interactions in natural cellulosome is a key step. Through structural predictions of protein interactions, such as the highly accurate prediction model AlphaFold 3 (Abramson et al., 2024), cohesin-dockerin affinities can be estimated without the need for laborious experiments. Future studies may leverage recent advances in protein structure prediction, such as AlphaFold 3, to explore cohesin-dockerin interactions in silico. In addition to predicting the 3D structures of protein complexes, AlphaFold 3 provides confidence metrics, including the predicted template modeling (pTM) score and predicted aligned error (PAE), which may help assess the reliability of predicted interactions (Abramson et al., 2024; Zhang and Skolnick, 2004; Varadi et al., 2022).

In addition, unique traits of fungal cellulosomes might be key to solving this. Low species-specificity of cohesins and dockerins can enhance compatibility between components, and additional C-terminal fusions on fungal dockerins allow capacity for more enzymes to be added to the cellulosome (Dorival et al., 2022).

#### 6.3.2 Improving thermostability of cellulosomal components

Due to the mesophilic origin of most cellulosomal components, the use of cellulosomes is often limited to temperatures up to 50°C (Bayer et al., 2004). To create a thermostable designer cellulosome with maintaining functional activity at elevated temperatures, one approach is to incorporate a scaffoldin composed of mesophilic modules with thermophilic enzymes (Galanopoulou et al., 2016). A tetravalent scaffoldin containing three mesophilic cohesins and one cohesin with a cellulose-binding module from *Clostridium thermocellum* was paired with thermophilic enzymes from *Geobacillus* and *Caldicellulosiruptor* species, each linked to dockerins matching the cohesins. This designer cellulosome maintained stability and functionality for at least 6 h at 60°C, achieving over 50% greater hydrolysis efficiency compared to free enzymes. The findings demonstrate that mesophilic scaffoldin components can be utilized at elevated temperatures, offering a viable strategy for designing cellulosomes for high-temperature biorefinery applications. Techniques such as thermostability enhancements and modular scaffoldin designs integrating mesophilic and thermophilic elements have enabled the creation of heat-resistant cellulosomes.

## 7 Conclusion

The utilization of lignocellulose biomass is a sustainable alternative to fossil carbon resources in producing biofuels to contribute to the circular economy. It is therefore of great interest to develop robust and efficient procedures to degrade lignocellulose. In this review we present the diverse lignocellulose degradation mechanisms found in nature, with the intention of using them as guidance for the development of more efficient degradation of biomass. Additionally, we highlight three key optimization focuses, including discovery of new species and enzymes, enzyme bioengineering and enhancing structural stability of designer cellulosomes. We expect these insights to serve as resources for the biomass community to develop more streamlined and sustainable solutions for utilizing lignocellulose as biofuel.

### 7.1 Future prospects

By drawing conclusions on lignocellulose degradation systems existing in nature, here a model favorable for industrial usage could be raised. After increasing the surface area of biomass using mechanical pre-treatment, lignin degradation is performed by bacterial AA enzymes to take advantage of their heat tolerance and ability to produce economical byproducts. Compared to costly thermochemical methods, pairing mechanical pretreatment and bacterial lignin degrading enzymes to remove recalcitrance could be more cost efficient. Recombinant expression of DyPs and laccase in *E. coli* have yielded high levels of expression (Lambertz et al., 2016; Ihssen et al., 2015), and bacterial cultivation benefits from fast growth rate and high tolerance to growth conditions. After lignin is removed, fungal endoglucanase should be used to degrade amorphous cellulose, followed by the application of designer cellulosomes to hydrolyze the remaining crystallized cellulose and hemicellulose. To capitalize on their cross-binding ability, designer cellulosomes are recommended to be assembled with fungal scaffoldin backbone and cohesin/dockerin modules. To ensure that the enzymes work synergistically, the arrangement of the enzymes on the cellulosome should take the natural succession of microorganism communities into consideration. For example, enzymes from white rot fungi should come before intermediate decay fungi followed by soft rot fungi. One of the challenges of designer cellulosome engineering lies in the large diversity of cellulosome components, creating exponential number of combinatorial possibilities that are costly to troubleshoot and optimize one by one (Lamote et al., 2023). Through advancements such as combinatorial assembly method via VersaTile platform (Vanderstraeten et al., 2022) and neural network models in protein–protein interaction prediction via AlphaFold 3 (Abramson et al., 2024), more economically efficient pipelines can be developed.

In the future, we anticipate answers to biological questions such as what is the mechanism behind the secretion of a selected choice of substrate-specific synergistic enzymes, how and when is the cellulosome assembled, what selection factors contribute to the repeated evolution of cellulosomal structure in both bacteria and fungi, etc. These insights, together with a wider collection of synthetic biology tools, will help actualize the usage of biomass as a sustainable energy.
